# Optimization of culture conditions for rapid clinical-scale expansion of human umbilical cord blood-derived mesenchymal stem cells

**DOI:** 10.1186/s40169-017-0168-z

**Published:** 2017-10-10

**Authors:** Wankyu Choi, Soon-Jae Kwon, Hye Jin Jin, Sang Young Jeong, Soo Jin Choi, Wonil Oh, Yoon Sun Yang, Hong Bae Jeon, Eun Su Jeon

**Affiliations:** Biomedical Research Institute, R&D Center, MEDIPOST Co., Ltd., 21 Daewangpangyo-ro 644beon-gil, Bundang-gu, Seongnam-Si, Gyeonggi-do 13494 Republic of Korea

**Keywords:** Calcium, Hypoxia, Human umbilical cord blood-derived mesenchymal stem cells, Clinical-scale culture, Cell therapy

## Abstract

**Background:**

Mesenchymal stem cells (MSCs) have broad-spectrum therapeutic effects in various diseases, and thus have many clinical applications. However, it is difficult to produce sufficient numbers of MSCs for clinical use, and improved culture systems are required. Here, we report the effects of calcium (Ca^2+^) and hypoxia on the proliferation of human umbilical cord blood-derived MSCs (hUCB-MSCs). In addition, we determined the optimal conditions of these two factors for the large-scale culture of hUCB-MSCs.

**Methods:**

hUCB-MSCs were maintained under hypoxic conditions (3% O_2_) with 1.8 mM Ca^2+^ during long-term culture, and their proliferation was evaluated. To characterize the underlying mechanisms, the effects on hypoxia-inducible factor (HIF)-1α and the extracellular signal-regulated kinase (ERK) signaling pathways were investigated. The therapeutic effects in a mouse emphysema model were analyzed and compared with those of naive MSCs.

**Results:**

The proliferation of Ca^2+^/hypoxia-treated hUCB-MSCs was increased compared with that observed using either calcium or hypoxia culture alone, without loss of stem cell marker expression or differentiation ability. The enhancement of the proliferation capacity of hUCB-MSCs by the synergistic effects of Ca^2+^ and hypoxia was dependent on the expression of HIF-1α and the ERK signaling pathway. The proliferation of Ca^2+^/hypoxia-treated hUCB-MSCs resulted in a delayed senescence phenotype and increased the expression levels of stemness genes such as *Oct4* and *Nanog* compared to those observed in conventional culture conditions. In addition, Ca^2+^/hypoxia-treated MSCs transplantation in the mouse emphysema model showed the same therapeutic effects as observed with naive MSCs.

**Conclusions:**

These findings suggest that a Ca^2+^/hypoxia-based expansion system has applications for the large-scale production of MSCs for therapeutic purposes.

**Electronic supplementary material:**

The online version of this article (doi:10.1186/s40169-017-0168-z) contains supplementary material, which is available to authorized users.

## Background

Human mesenchymal stem cells (hMSCs), which are capable of self-renewal and differentiation into various mesenchymal tissues, including osteogenic, adipogenic, and chondrogenic cell lineages, have been recognized as a promising tool for clinical applications [1, 2]. Human umbilical cord blood-derived MSCs (hUCB-MSCs) have a high rate of cell proliferation, low senescence, and extensive anti-inflammatory effects, suggesting that these cells have high therapeutic utility [1, 2]. The secretion of soluble factors with autocrine and paracrine actions is one of the key mechanisms underlying the therapeutic potential of MSCs [3–7], and hUCB-MSCs have recently been shown to have therapeutic efficacy in various disease models [8–11]. However, the therapeutic potential of hMSCs cannot be realized without gaining a more complete understanding of the in vitro culture parameters that can best maintain the stem cell phenotype and multipotency during expansion. Moreover, it is difficult to obtain the large numbers of cells required for MSC clinical applications (i.e., up to 5 × 10^6^ MSCs/kg body weight) with conventional culture methods [12]. Thus, the development of novel, simple culture methods for obtaining large numbers of hUCB-MSCs is necessary.

Many factors have been reported to increase the proliferation of MSCs. Among these factors, hypoxia is known to regulate several cellular processes, including proliferation, survival, and differentiation [13, 14]. Indeed, the use of hypoxia (3% O_2_) during the isolation step has been shown to select for a highly primitive hMSC-like cell population from the bone marrow with markedly improved self-renewal abilities [15]. In addition, hypoxic culture conditions were shown to affect the therapeutic properties of hMSCs [16]. Calcium (Ca^2+^) is a ubiquitous intracellular signal responsible for regulating numerous cellular processes such as cell differentiation, proliferation, apoptosis, and secretion. In MSCs, elevated Ca^2+^ has been reported to stimulate cell proliferation and differentiation, which are important functions of stem cells [17, 18]. Thus, combined treatment with Ca^2+^ and hypoxia may enhance the growth of MSCs and inhibit the decrease in proliferation capacity caused by repeated subculturing. However, the effects of the combination of Ca^2+^ and hypoxia on the expansion of hMSCs have not been elucidated to date.

Therefore, in this study, we examined whether combined Ca^2+^/hypoxia would promote the proliferation of hUCB-MSCs and maintain the MSC phenotype during monolayer culture. To determine the underlying mechanism, we further evaluated the influence of Ca^2+^/hypoxia on hypoxia-inducible factor-1 alpha (HIF-1α) and the extracellular-related signal kinase (ERK) pathway on the induced proliferation of hUCB-MSCs. These markers were chosen since HIF-1α plays a key role in the cellular response to hypoxia by activation of various genes involved in proliferation of MSCs [19]. Furthermore, we investigated whether Ca^2+^/hypoxia-treated hUCB-MSCs show the same therapeutic effects compared with hUCB-MSCs cultured under standard conditions using an in vivo emphysema animal model.

Our results are expected to provide important insights into the optimization of culture methods to promote the clinical-scale expansion of hMSCs.

## Methods

### Materials

Alpha-minimum essential medium (MEM) and fetal bovine serum (FBS) were purchased from Gibco (Carlsbad, CA, USA). Trypsin, phosphate-buffered saline (PBS), and distilled water were purchased from Biowest (Carlsbad, CA, USA). Lipofectamine 3000 reagent was purchased from Invitrogen (Carlsbad, CA, USA). The extracellular signal-regulated kinase (ERK) inhibitor U0126, phospho-ERK antibody, total ERK antibody and peroxidase-labeled secondary antibodies were purchased from Cell Signaling Technology (Beverly, MA, USA). Antibodies for hypoxia-inducible factor (HIF)-1α were purchased from BD Biosciences (Oxford, UK). Antibodies for GAPDH were provided by the Gwangju Institute of Science and Technology, Korea (GIST). The intracellular calcium chelatorBAPTA-AM was purchased from Calbiochem (La Jolla, CA, USA).

### Isolation of hUCB-MSCs and culture conditions

hUCB-MSCs were collected from the umbilical cord vein of a newborn baby, with the consent of the mother. To isolate and expand MSCs from cord blood, mononuclear cells (MNCs) were isolated using a Ficoll–Hypaque solution (*d* = 1.077 g/cm^3^; Sigma). The cells were then seeded at 5 × 10^5^ cells/cm^2^ in culture flasks. After colonies of spindle-shaped cells were formed, the cells were reseeded for expansion. hUCB-MSCs were cultured in αMEM medium supplemented with 10% FBS and gentamycin in a humidified atmosphere containing 5% CO_2_ and various concentrations of oxygen (3 or 21%) at 37 °C.

### Cell proliferation assay

hUCB-MSC proliferation was evaluated using a BrdU incorporation assay kit (Amersham; Cell Proliferation Biotrak ELISA System; RPN250; GE Healthcare, Little Chalfont, UK) according to the manufacturer instructions. The population doubling (PD) number was calculated based on the total cell number at each passage. PD was calculated at every passage by dividing the logarithm of the fold increase value obtained at the end of the passage by the logarithm of 2. This procedure was repeated until the cells stopped proliferating. At this point, the number of cells was counted to calculate the final PD. The population doubling time (PDT) was determined using the following formula: (*t* − *t*_0_) × log2/log(*N* − *N*_0_), where *t* − *t*_0_ is culture time (h), *N* is the number of harvested cells, and *N*_0_ is the number of initial cells [1].

### Emphysema model

Female C57BL/6J mice (18–20 g, 7 weeks of age) were intratracheally injected with 0.4 U of pancreatic elastase (Sigma-Aldrich, St. Louis, MO, USA) at day 0, intravenously injected through the tail vein with 2 × 10^4^ of MSCs at day 7, and sacrificed at day 14. The lungs were perfused with phosphate buffered saline and inflated by intratracheal infusion of 0.5% low-melting agar at 25 cm H_2_O, fixed in 4% paraformaldehyde, and embedded in paraffin. Lung sections of 4-µm thickness were stained with hematoxylin and eosin. To evaluate the extent of alveolar destruction, the mean linear intercepts (MLIs) were determined separately by two investigators in a blinded fashion.

### Western blot analysis

For western blot analysis, hUCB-MSCs were washed with ice-cold 1 × PBS and lysed with RIPA buffer containing protease inhibitors and phosphatase inhibitors (Roche). Protein concentrations were determined by the Bradford assay. Lysates were separated using Novex, NuPAGE, and Bolt precast gels (Invitrogen) under denaturing conditions and transferred to nitrocellulose membranes. After blocking with 1% bovine serum albumin (BSA) solution, membranes were immunoblotted with various antibodies and then probed with horseradish peroxidase-conjugated secondary antibodies. Protein bands were visualized using an enhanced chemiluminescence (ECL) immunoblotting system (GE Healthcare).

### Real-time polymerase chain reaction (PCR)

Total RNA was extracted from cell pellets using TRIzol reagent (Invitrogen). cDNA was synthesized using 5 g of total RNA, 200 U of SuperScript III reverse transcriptase (Invitrogen), and 0.5 g oligo (dT) 15 primer (Promega, Madison, WI, USA). For real-time PCR, the amplification was performed in a total volume of 20 µL containing LightCycler universal probe and LightCycler TaqMan Master Mix using a LightCycler 480 Real-Time PCR System (Roche). PCR assays were performed in triplicate with the primer sequences shown in Table 1, and the PCR protocol was as follows: 95 °C for 10 min, followed by 45 cycles of denaturation at 95 °C for 10 s, annealing at 62 °C for 30 s, and extension at 72 °C for 10 s. Expression levels of genes were normalized to those of the endogenous control (*GAPDH*).

**Table 1:** Primer sequences

Gene	Primer sequence
*GAPDH*	F: 5′-AGCCACATCGCTCAGACAC-3′
	R: 5′-GCCCAATACGACCAAATCC-3′
*p16*	F: 5′-GTGGACCTGGCTGAGGAG-3′
	R: 5′-CTTTCAATCGGGGATGTCTG-3′
*p53*	F: 5′-CCCCAGCCAAAGAAGAAAC-3′
	R: 5′-AACATCTCGAAGCGCTCAC-3′
*p21*	F: 5′-CGAAGTCAGTTCCTTGTGGAC-3′
	R: 5′-CATGGGTTCTGACGGACAT-3′
*Nanog*	F: 5′-AGATGCCTCACACGGAGACT-3′
	R: 5′-TTTGCGACACTCTTCTCTGC-3′

### Multi-lineage differentiation analysis

To induce osteogenic differentiation, the cells were seeded at a density of 5 × 10^2^ cells/cm^2^ and cultured in 6-well plates for 3 weeks in osteogenic differentiation medium. The induction medium was alpha-minimal essential medium (α-MEM) supplemented with 0.1 µM dexamethasone, 10 mM β-glycerolphosphate, 0.05 mM l-ascorbic acid, and 10% fetal bovine serum (FBS) (Gibco) [20, 21]. The medium was changed twice a week. The onset of osteoblast formation was evaluated by analyzing the expression of alkaline phosphatase (ALP).

To promote chondrogenic differentiation, 2 × 10^5^ cells were gently centrifuged in a 15-ml polypropylene tube to form a pellet micromass. The micromass was then treated with chondrogenic medium for 4 weeks. The medium was replaced twice a week. The induction medium consisted of high-glucose Dulbecco’s modified Eagle medium supplemented with 0.1 µM dexamethasone, 50 µg/mL l-ascorbic acid, 0.1 mg/ml sodium pyruvate, 40 µg/mL l-proline, 10 ng/mL transforming growth factor-beta 3, 0.5 µg/mL bone morphogenic protein-6, and 50 mg/mL ITS^+^ premix [20, 22]. After the culture period, cryosections were analyzed by safranin O staining. The sections were fixed with 95% ethanol and stained with 0.1% aqueous safranin O solution.

To induce adipogenic differentiation, the cells were treated with adipogenic medium [α-MEM supplemented with 10% FBS (Gibco), 0.5 mm 3-isobutyl-1-methylxanthine (Sigma), 1 mm dexamethasone (Sigma), 0.2 mm indomethacin (Sigma), and 10 mm h-insulin (Sigma) [20] for 4 weeks. Medium changes were carried out twice a week. The onset of adipocytes was evaluated with Oil-red O (Sigma) stain.

### Senescence-associated β-galactosidase (SA-β-gal) activity assays

SA-β-gal staining was conducted after the seventh subculture. The assay is based on detection of β-galactosidase at pH 6 using a senescence β-galactosidase staining kit (Cell Signaling Technology). Cells were washed once with PBS, fixed in the fixative solution, and incubated in the staining solution overnight. Cells were examined on an inverted microscope. To assess senescent-cell formation, the overall percentage of stained cells in the cell populations was averaged from four fields.

### Flow cytometry

For cytometric analysis of cultured cell phenotypes, the cells were stained with antibodies against human CD14, CD34, CD45, HLA-DR (FITC, BD Biosciences), CD73, CD90 (PE, Pharmingen, Los Angeles, CA, USA), CD166 (PE, BD Biosciences), and CD105 (PE, Serotec, Kidlington, UK) for 15 min at room temperature. Corresponding mouse isotype antibodies were used as controls. The cells were then washed twice with DPBS and fixed 1% paraformaldehyde (PFA). At least 10,000 events were measured using a fluorescence-activated cell sorting instrument (FACSCalibur; Becton–Dickinson, San Jose, CA, USA), and cell flow cytometry data were analyzed using CELLQUEST software (Becton–Dickinson).

### Transfection with small-interfering RNA (siRNA)

RNA interference (RNAi) oligoribonucleotides were synthesized, desalted, and purified by ST Pharm. Co. Ltd. (Siheung, Gyeonggi, Korea) with the sequences shown in Table 2. For siRNA experiments, hUCB-MSCs were transfected with 100 nM HIF-1α or control siRNAs using lipofectamine 3000 reagent (Invitrogen) according to the manufacturer’s instructions. Cells were cultured in growth medium for 48 h, and the knockdown efficiencies of the target genes were confirmed by determining the decrease in the expression level of total HIF-1α.

**Table 2:** siRNA target sequences

Gene	Si RNA sequence
Nonspecific control	F: 5′-GAGAAAUGGUGCGAGAAGdTdT-3′
	R: 5′-CUUCUCGCACCAUUUCUCCdTdT-3′
HIF-1α	F: 5′-GUCCCAUGAAAAGACUUAAdTdT-3′
	R: 5′-UAAGUCUUUUCAUGGGACdTdT-3′

### Statistical analysis

All data are reported as mean ± standard deviation (SD) and were analyzed by SPSS (version 18, SPSS Inc., Chicago, IL, USA). Differences and significance were verified by one-way ANOVA followed by the Fisher’s least significant difference (LSD) post hoc test. *P* values < 0.05 were considered statistically significant.

## Results

### Optimization of culture conditions for increasing the proliferation capacity of hUCB-MSCs

**Fig. 1:**
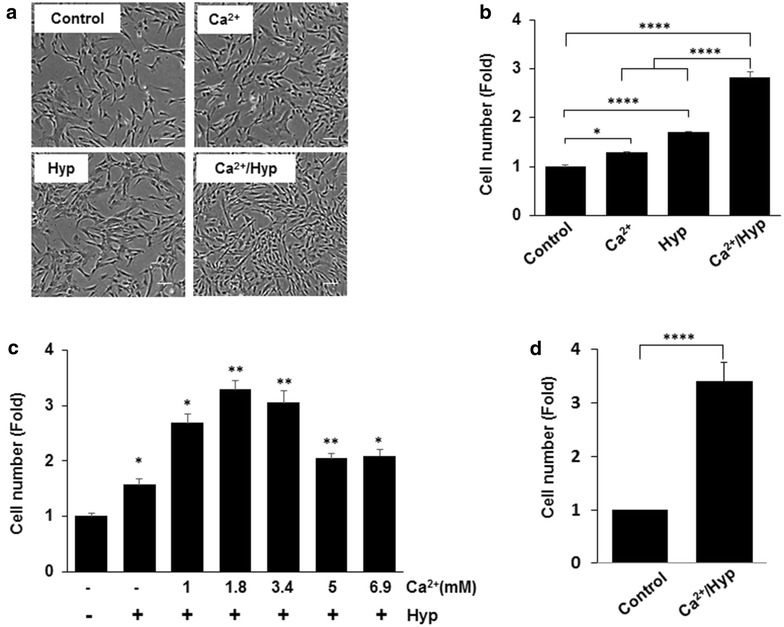
Effects of Ca^2+^/hypoxia on the proliferation capacity of hUCB-MSCs. **a**, **b** hUCB-MSCs were treated with Ca^2+^, hypoxia, or Ca^2+^/hypoxia for 5 days. The morphology and growth curve of cultured hUCB-MSCs were analyzed. Ca^2+^/Hyp: Ca^2+^ plus hypoxia; Hyp: hypoxia. **c** hUCB-MSCs were treated with the indicated concentrations of Ca^2+^ under hypoxic conditions (3% oxygen) for 5 days. **d** Six batches of hUCB-MSCs were tested for proliferation in the Ca^2+^/hypoxia condition. The initial cell concentration of hUCB-MSCs was 5 × 10^3^ cells/cm^2^. Cell proliferation was measured by cell counting assays. Data represent the mean ± SD, *n* = 3; *P < 0.05, **P < 0.01, ***P < 0.001, ****P < 0.0001. Scale bar = 100 μm (magnification: 100 ×)

To determine whether the modification of culture conditions can stimulate cell proliferation, hUCB-MSCs were treated with Ca^2+^, hypoxia, or combined Ca^2+^/hypoxia. We determined the cell proliferation of hUCB-MSCs by the cell counting method after the indicated treatments were applied for 5 days. As shown in Fig. 1a, b, Ca^2+^/hypoxia conditioning synergistically enhanced the proliferation capacity of hUCB-MSCs compared to cells cultured with Ca^2+^ or hypoxia-only (combined Ca^2+^/hypoxia: 2.8-fold, Ca^2+^: 1.2-fold, and hypoxia: 1.7-fold compared to the control). Under the hypoxic condition (3% O_2_), treatment with Ca^2+^ increased the proliferation of the cells in a concentration-dependent manner, with a maximal effect observed at 1.8 mM Ca^2+^ (Fig. 1c). As shown in Fig. 1d, six batches of hUCB-MSCs were tested in the Ca^2+^/hypoxia condition, and the growth of these hUCB-MSCs was found to have increased by approximately 3.5-fold as compared to naïve hUCB-MSCs. The use of six different batches minimized the potential effect of batch-to-batch variation.

### Ca^2+^/hypoxia enables hUCB-MSCs to maintain their synergistic proliferation capacity in long-term culture

**Fig. 2:**
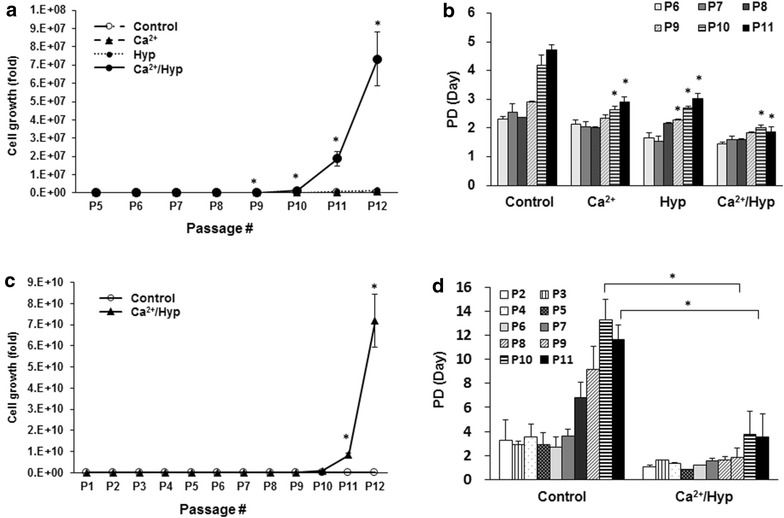
Ca^2+^/hypoxia-treated hUCB-MSCs maintained their proliferation capacity in long-term culture. **a**, **b** Growth curves and population doubling (PD) time of hUCB-MSCs in individual treatments were analyzed for all passages during long-term culture. **c**, **d** After isolation of MNCs from UCB, the cells were maintained under the two culture conditions. Growth curves and PD time were analyzed for hUCB-MSCs treated with Ca^2+^/hypoxia through a series of passages. The initial cell concentration of hUCB-MSCs was 5 × 10^3^ cells/cm^2^. Cell proliferation was measured by cell counting assays. Data represent the mean ± SD, *n* = 3; *P < 0.05 versus control

We evaluated the synergistic proliferation of hUCB-MSCs with and without Ca^2+^/hypoxia treatment through a series of passages. Figure 2a shows that the culture under combined Ca^2+^/hypoxia treatment enhanced the total growth of hUCB-MSCs by approximately 4000-fold over 7 weeks, which was much greater than the increases observed under the Ca^2+^ or hypoxia culture condition alone (Ca^2+^: 28-fold, and hypoxia: 86-fold compared to the control). The doubling time of hUCB-MSCs in individual treatments increased during repeated sub-cultures, but Ca^2+^/hypoxia conditions enabled the hUCB-MSCs to maintain their proliferation capacity in long-term culture (Fig. 2b). Thus, we applied this method to immediately isolate the MSCs from the UCB. After isolation of MNCs from the UCB, the cells were cultured under normal or Ca^2+^/hypoxia culture conditions (Fig. 2c). hUCB-MSCs isolated with Ca^2+^/hypoxia exhibited 2.7 × 10^5^-fold greater total growth at passage 12 than hUCB-MSCs isolated under normal culture conditions. The doubling time was also lower in the Ca^2+^/hypoxia condition during long-term culture (Fig. 2d).

### Ca^2+^/hypoxia enhanced the proliferation of hUCB-MSCs by upregulating HIF-1α

**Fig. 3:**
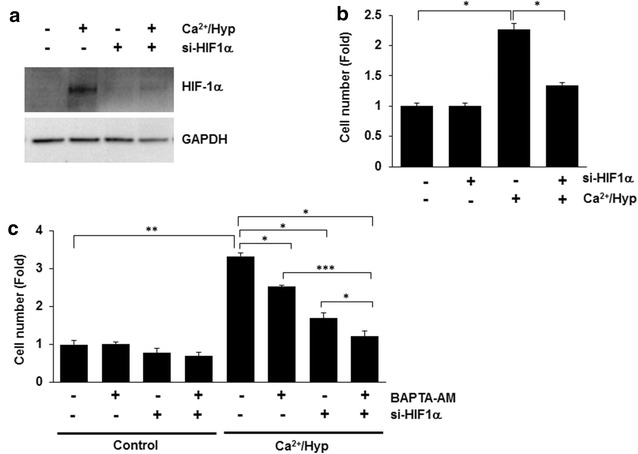
Ca^2+^/hypoxia stimulated the proliferation of hUCB-MSCs via the HIF-1α-dependent pathway. **a** At 48 h post-transfection with control siRNA or HIF-1α-specific siRNA (si-HIF-1α), hMSCs were treated with Ca^2+^/hypoxia for 6 h. The expression of HIF-1α was analyzed by western blot analysis. **b** After growth under the Ca^2+^/hypoxia condition for 5 days, the proliferation capacity of HIF-1α-knockdown cells was measured by cell counting assays. **c** hUCB-MSCs were transfected with HIF-1α siRNA, pretreated with vehicle or 5 μM BAPTA-AM for 30 min, and then treated with Ca^2+^/hypoxia for 5 days. The proliferation capacity was determined by cell counting assays. Data represent the mean ± SD, *n* = 3; *P < 0.05, **P < 0.01, ***P < 0.001

HIF-1α expression was determined by western blot analysis. As shown in Fig. 3a, HIF-1α expression in hUCB-MSCs increased following culture in the presence of Ca^2+^ and hypoxia. Therefore, we next examined the effects of HIF-1α knockdown by treatment of hUCB-MSCs with siRNA, followed by culture under Ca^2+^/hypoxic conditions for 6 h. We then examined whether HIF-1α was involved in the Ca^2+^/hypoxia-dependent enhancement of the hUCB-MSC proliferation capacity. In the absence of HIF-1α, Ca^2+^/hypoxia did not enhance the proliferation of hUCB-MSCs (Fig. 3b). These results suggest that Ca^2+^/hypoxia enhanced the proliferation capacity of hUCB-MSCs through an HIF-1α-dependent signaling pathway. Furthermore, pretreatment with BAPTA-AM, an intracellular calcium chelator, in si-HIF-1α-transfected cells treated with Ca^2+^/hypoxia completely blocked cell proliferation (Fig. 3c).

### ERK was involved in the Ca^2+^/hypoxia-mediated enhancement of proliferation capacity in hUCB-MSCs

**Fig. 4:**
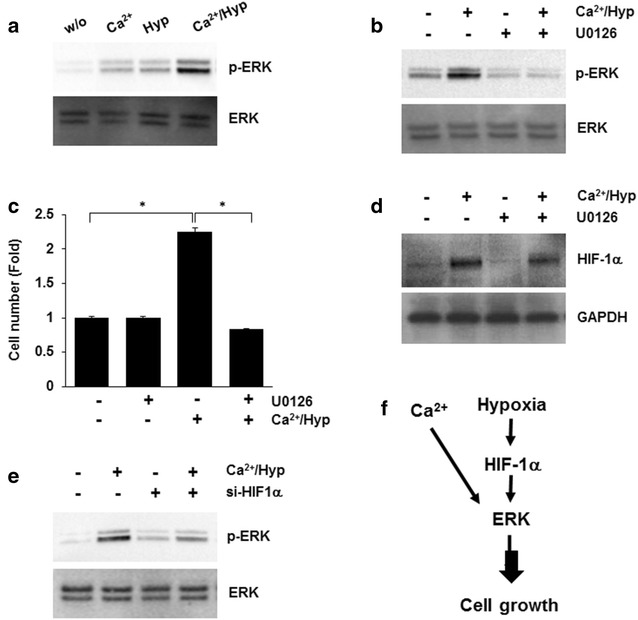
The role of ERK in the Ca^2+^/hypoxia-mediated enhancement of hUCB-MSC proliferation. **a** hMSCs were treated with Ca^2+^/hypoxia and then harvested after 30 min. The phosphorylation of ERK was determined by western blot analysis. **b** hMSCs were treated with Ca^2+^/hypoxia for 30 min in the absence or presence of U0126. The phosphorylation of ERK was analyzed by western blot analysis. **c** hUCB-MSCs were pretreated with vehicle or U0126 for 30 min and then exposed to Ca^2+^/hypoxia for 5 days. Proliferation capacity was determined by cell counting assays. **d** hMSCs were treated with Ca^2+^/hypoxia for 6 h in the absence or presence of U0126. HIF-1α expression was determined by western blot analysis. **e** At 48 h post-transfection with HIF-1α-specific siRNA (si-HIF-1α), hMSCs were cultured under Ca^2+^/hypoxia for 30 min. The phosphorylation of ERK was determined by western blot analysis. **f** Schematic illustration of the molecular mechanisms involved in Ca^2+^/hypoxia-induced cell proliferation. Data represent the mean ± SD, *n* = 3; *P < 0.01

Ca^2+^ and hypoxia have been reported to induce ERK activation [23, 24]. Therefore, to elucidate the role of ERK in the Ca^2+^/hypoxia-mediated enhancement of the proliferation capacity in hUCB-MSCs, hUCB-MSCs were cultured with Ca^2+^, hypoxia, or Ca^2+^ plus hypoxia for 30 min, and the phosphorylation of ERK was analyzed by western blotting (Fig. 4a). As shown in Fig. 4b, Ca^2+^/hypoxia induced ERK phosphorylation, and this effect was prevented by pretreatment with U0126, a specific inhibitor of MEK1/2. In addition, pretreatment with U0126 significantly inhibited Ca^2+^/hypoxia-induced proliferation (Fig. 4c). By contrast, the Ca^2+^/hypoxia-induced expression of HIF-1α was not changed by pretreatment with U0126 (Fig. 4d). Next, hUCB-MSCs were transfected with control or HIF-1α siRNA and then cultured under Ca^2+^/hypoxic conditions. ERK phosphorylation was blocked by HIF-1α silencing (Fig. 4e). These results suggest that combined treatment with Ca^2+^ and hypoxia induced the synergistic proliferation of hUCB-MSCs through an HIF-1α/ERK-dependent signaling pathway (Fig. 4f).

### Ca^2+^/hypoxia enhanced proliferation without decreasing stemness

**Fig. 5:**
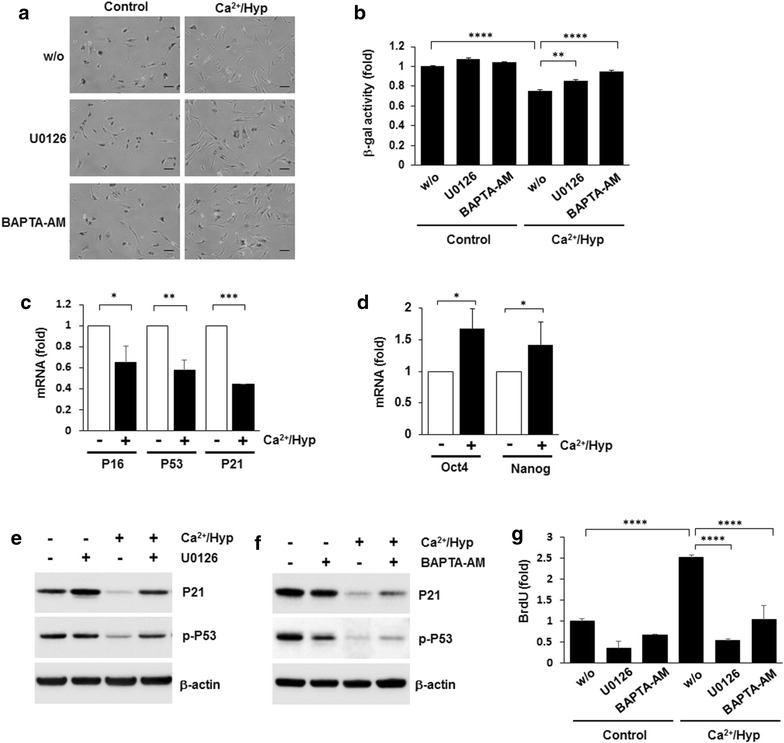
Effects of Ca^2+^/hypoxia on the senescence of hUCB-MSCs. hMSCs were treated with Ca^2+^/hypoxia for five passages in the absence or presence of U0126 or BAPTA-AM. **a** Senescence stage was determined using SA-β-gal activity assays. **b** Staining was quantified by positive cell counts. **c**, **d** The mRNA levels of senescence-associated genes, i.e., p16, p53, and p21, or stemness marker genes, i.e., Oct4 and Nanog, were determined by real-time PCR analysis. **e**, **f** The expression levels of senescence-associated proteins, i.e., p21 and p-p53, were determined by western blot analysis. **g** Ca^2+^/hypoxia-induced cell proliferation was significantly decreased in the groups treated with U0126 or BAPTA-AM. Data represent the mean ± SD, n = 3; #*P* < 0.01, *P < 0.05, **P < 0.01, ***P < 0.001, ****P < 0.0001. Scale bar = 100 μm (magnification: 100 ×)

We also evaluated the induction of senescence in these cells by analyzing the expression patterns of senescence and stemness marker genes. The hUCB-MSCs were subjected to SA-β-gal activity assays. Senescence progression was decreased in Ca^2+^/hypoxia-treated hUCB-MSCs as compared to that in hUCB-MSCs cultured under normal conditions. In addition, pretreatment with U0126 or BAPTA-AM significantly inhibited Ca^2+^/hypoxia-delayed senescence (Fig. 5a, b). The senescence marker genes *p16*, *p53*, and *p21* were downregulated, and the stemness marker genes *OCT4* and *NANOG* were upregulated in hUCB-MSCs treated with Ca^2+^/hypoxia (Fig. 5c, d). In addition, pretreatment with U0126 or BAPTA-AM significantly inhibited the Ca^2+^/hypoxia-reduced p21 expression and p53 phosphorylation levels (Fig. 5e, f). Furthermore, Ca^2+^/hypoxia-treated hUCB-MSCs showed increased BrdU incorporation in an ERK-dependent manner (Fig. 5g). These results suggest that Ca^2+^/hypoxia culture enhanced the proliferation capacity without decreasing the stemness of the hUCB-MSCs.

### Ca^2+^/hypoxia-conditioned hUCB-MSCs maintained stem cell phenotypes and therapeutic effects

**Fig. 6:**
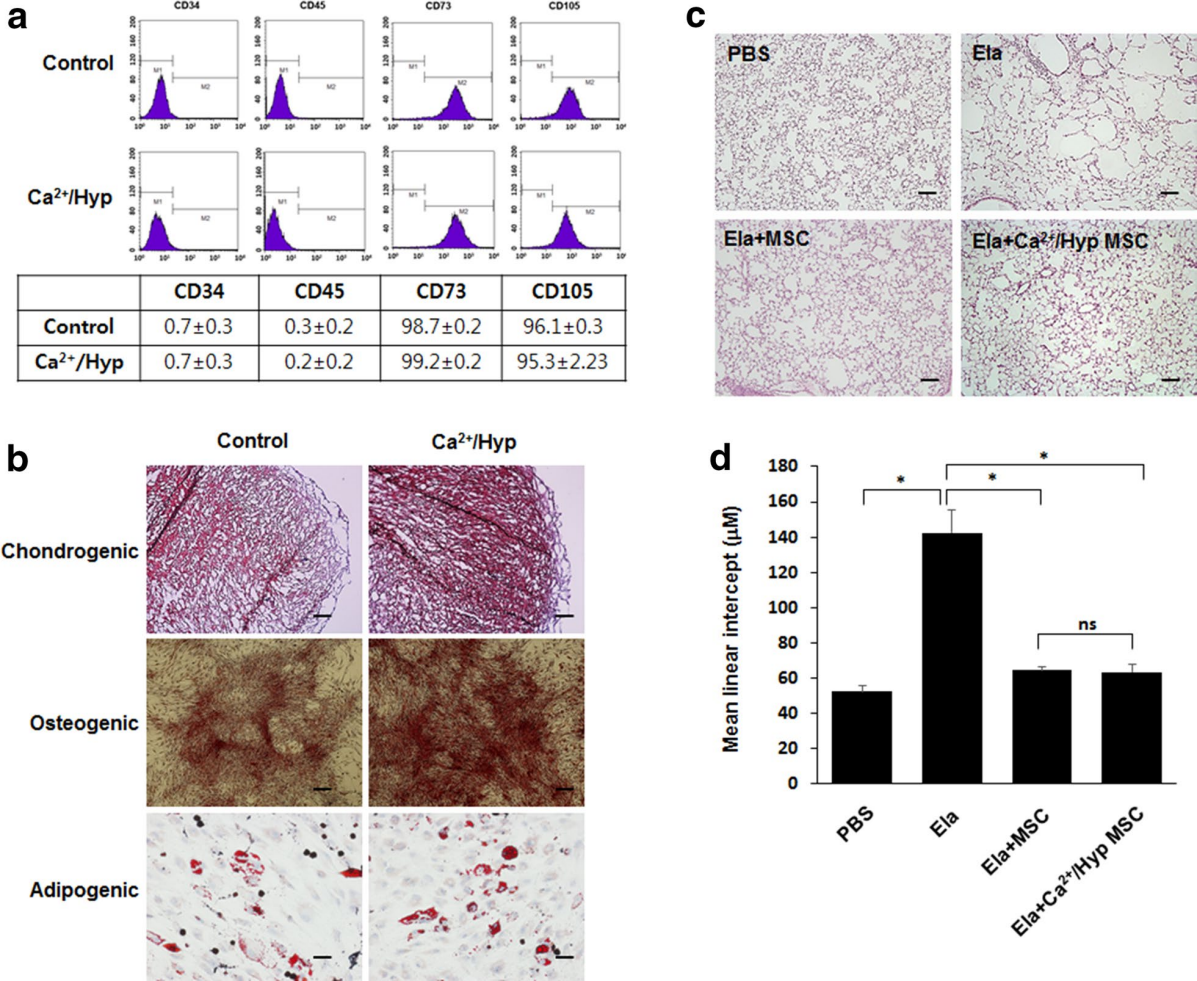
Effects of Ca^2+^/hypoxia on the stem cell phenotypes of hUCB-MSCs. **a** Immunophenotypic analysis of CD34, CD45, CD73, and CD105 expression in naïve or Ca^2+^/hypoxia-conditioned hUCB-MSCs was performed using flow cytometry. **b** After incubation in specialized induction media, chondrogenic differentiation was measured by the accumulation of sulfated proteoglycan using safranin O staining (top), and osteogenic differentiation was evaluated by alkaline phosphatase staining (bottom). Data represent the mean ± SD (n = 3). *P < 0.05 versus the control as determined by Student’s t-tests. **c** The therapeutic effects of hUCB-MSCs in an elastase-induced emphysema model. Representative histological image of the lung parenchyma stained with hematoxylin-eosin (H&E). **d** Morphometric analysis of the mean linear intercept. Data represent the mean ± SD, *n* = 3; *P < 0.0001. Scale bar = 100 μm (magnification: 100 ×)

We investigated the effects of Ca^2+^/hypoxia treatment on the stem cell properties of hUCB-MSCs by measuring the expression of MSC-specific markers with FACS analysis [1]. Ca^2+^/hypoxia-treated hUCB-MSCs expressed MSC-specific markers, including CD73 and CD105. Notably, these cells were negative for CD34 and CD45 expression (Fig. 6a and Additional file 1: Figure S1). Next, the effects of Ca^2+^/hypoxia treatment on the chondrogenic and osteogenic differentiation of hUCB-MSCs were investigated. As shown in Fig. 6b, Ca^2+^/hypoxia-treated hUCB-MSCs showed no significant difference in differentiation as compared to naïve hUCB-MSCs. We also confirmed the genetic stability by conducting chromosomal karyotype analysis of Ca^2+^/hypoxia-treated hUCB-MSC (Additional file 2: Figure S2). To identify any difference of naïve hUCB-MSC and Ca^2+^/hypoxia-conditioned hUCB-MSCs for the treatment of emphysema, C57BL/6J mice were treated intratracheally with 0.4 U of elastase on day 0 and were intravenously injected with 2 × 10^4^ of MSCs on day 7. The lung tissue was then collected on day 14. The mice treated with elastase-only showed severe alveolar destruction compared to the control group, whereas the mice treated with MSCs showed relatively less alveolar destruction (Fig. 6c). The level of alveolar destruction was quantified by measuring the MLI values. The MLI decreased in mice treated with the naïve hUCB-MSCs or Ca^2+^/hypoxia-conditioned hUCB-MSCs (64.49 ± 1.72 µm or 63.32 ± 4.39 µm, respectively) compared to those treated with elastase only (142.58 ± 12.86 µm), but the differences were not statistically significant (Fig. 6d).

## Discussion

The clinical demand for MSCs has driven the need for the development of robust, large-scale production techniques. However, the limited proliferation capacity and senescence tendency of MSCs during expansion have thus far limited their clinical applications. Common cell culture techniques enable the billion-fold expansion of hMSCs [19] but also cause the gradual loss of their primitive characteristics and self-renewal properties. In the present study, we sought to overcome this major obstacle for the large-scale production of MSCs for clinical use, and demonstrated the utility of a combination of Ca^2+^ and hypoxia for the expansion of hUCB-MSCs. In general, hMSCs are cultured in vitro in an atmosphere containing 21% oxygen tension. However, studies have demonstrated that the physiological niches from which hMSCs are isolated in the human body have an oxygen tension much lower than 21% [25, 26], as the physiological condition of most tissues is ~ 3–5% oxygen [27]. Importantly, in this study, after an initial period of adaptation to hypoxic conditions, the proliferation rate of hUCB-MSCs was found to be higher in 3% O_2_ than in 21% O_2_. Thus, our results provide important insights into the optimal culture conditions for the large-scale expansion of hUCB-MSCs.

Moreover, Ca^2+^ metabolism plays an important role in the control of cellular proliferation. Several studies investigating increased Ca^2+^ concentrations in cell culture medium have shown that Ca^2+^ enhances cell proliferation in a concentration-dependent manner, although high Ca^2+^ levels may induce cytosolic calcium overload and result in spontaneous apoptosis [23]. However, the effects of Ca^2+^/hypoxia on the expansion of hMSCs had not yet been elucidated. The results of the present study show that Ca^2+^/hypoxia treatment yielded approximately 4000-fold higher hUCB-MSC expansion over 7 weeks, whereas Ca^2+^ or hypoxia alone increased proliferation by only 28- and 86-fold, respectively. Importantly, we applied the Ca^2+^/hypoxia combined-culture method immediately after isolation of MNCs from the UCB. This treatment resulted in a 2.7 × 10^5^-fold growth increase as compared to growth under general normoxic culture conditions. We also confirmed that the addition of Ca^2+^/hypoxia in the initial MNC culture does not affect the efficacy in MSCs colony isolation (data not shown). Thus, these findings suggest that the Ca^2+^/hypoxia combined-culture method is extremely useful for the large-scale culture of hUCB-MSCs for therapeutic purposes after isolation of MNCs from the UCB.

Hypoxia activates mitogen-activated protein kinase (MAPK) and other signaling pathways. Moreover, hypoxia has been shown to enhance proliferation through an ERK-dependent pathway [28]. Calcium also activates MAPK signaling pathways, thereby regulating cell growth [29]. In the present study, hypoxia increased cell proliferation through HIF-1α-dependent ERK activation. Furthermore, under hypoxic conditions, treatment with Ca^2+^ induced synergistic proliferation through stimulation of hypoxia-induced ERK activation. Thus, Ca^2+^/hypoxia treatment enhanced the growth of hUCB-MSCs and maintained their proliferation capacity in long-term culture through an HIF-1α/ERK-dependent pathway.

The hyperproliferative response can be followed by cellular senescence [30]. Cellular senescence mechanisms are partly involved in the decline of stem cell activity in aging, and thus late-passage MSCs are often less effective than early-passage MSCs [31, 32]. Importantly, the combination of Ca^2+^ and hypoxia synergistically increased the expansion of hUCB-MSCs, without affecting their proliferation capacity or stem cell capability in long-term culture. In addition, we showed that the stemness genes were upregulated, whereas the senescence marker genes were downregulated in Ca^2+^/hypoxia-conditioned hUCB-MSCs. Our results further demonstrated that Ca^2+^/hypoxia-treated MSCs transplantation had the same therapeutic effects as transplantation of naive MSCs in an emphysema model.

## Conclusions

In conclusion, we demonstrate that the Ca^2+^/hypoxia-combined culture method had synergistic effects on cell proliferation in hUCB-MSCs. These findings suggest that culture of hUCB-MSCs with Ca^2+^/hypoxia facilitates the large-scale production of MSCs for therapeutic purposes.

## Additional files

**Additional file 1: Figure S1.** Flow cytometric characterization of naïve hUCB-MSC (control) and Ca^2+^/hypoxia-treated hUCB-MSC (Ca^2+^/Hyp).

**Additional file 2: Figure S2.** Karyotyping analysis of naïve hUCB-MSC (control) and Ca^2+^/hypoxia-treated  hUCB-MSC (Ca^2^^+^/Hyp).

## Abbreviations

hMSCs: human mesenchymal stem cells
HIF-1α: hypoxia-inducible factor-1α
hUCB-MSCs: human umbilical cord blood-derived MSCs
ERK: extracellular signal-regulated kinase
FBS: fetal bovine serum
PBS: phosphate-buffered saline
MNCs: mononuclear cells
MLIs: mean linear intercepts
