# Supplementary figures and images for: Optimization of culture conditions for rapid clinical-scale expansion of human umbilical cord blood-derived mesenchymal stem cells
Source: Clin Transl Med. 2017 Oct 10;6:38. doi: 10.1186/s40169-017-0168-z (PMC5634990; doi:10.1186/s40169-017-0168-z)

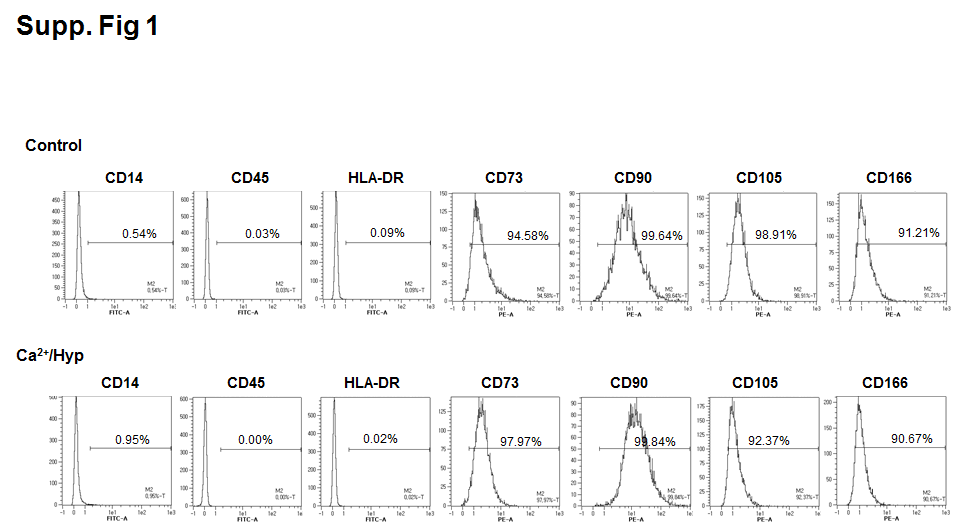

Supplement: Supplementary file 1 — Additional file 1: Figure S1. Flow cytometric characterization of naïve hUCB-MSC (control) and Ca2+/hypoxia-treated hUCB-MSC (Ca2+/Hyp). [file 40169_2017_168_MOESM1_ESM.tif]

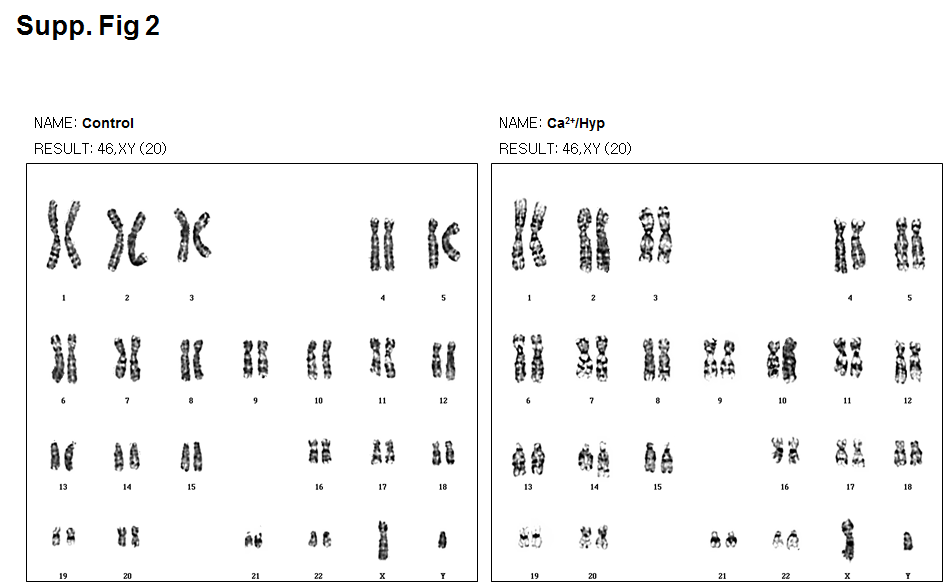

Supplement: Supplementary file 2 — Additional file 2: Figure S2. Karyotyping analysis of naïve hUCB-MSC (control) and Ca2+/hypoxia-treated hUCB-MSC (Ca2 +/Hyp). [file 40169_2017_168_MOESM2_ESM.tif]
